# Supplementary material for: Inferring genotype-phenotype maps using attention models
Source: ArXiv. 2025 Apr 14:arXiv:2504.10388v1. Preprint. [Version 1] (PMC12047939)
Supplement: Supplement 1 [file NIHPP2504.10388v1-supplement-1.pdf]

The code used in this work is available at our GitHub repository https://github.com/Emergent-Behaviors-in-Biology/GenoPhenoMapAttention. We implement our models using the PyTorch framework [39]. In the following sections, we detail the procedures and numerical parameters used to generate synthetic data, implement the attention-based models, and perform transfer learning.

Appendix A: Filtering loci for reduced correlation

Genetic variants at different loci can be correlated due to linkage disequilibrium, where nearby SNPs tend to be inherited together. To account for this, we first shuffle the genotype data and split it into training (85%) and test (15%) datasets. Next, we compute a correlation matrix C, a symmetric matrix where each entry $C_{i,j}$ represents the correlation coefficient between the i-th and j-th SNPs across individuals in the training dataset. The matrix has dimensions $41,594 \times 41,594$, and its values range from -1 (perfect negative correlation) to 1 (perfect positive correlation). The correlation matrix is computed as:

$$C_{i,j} = \frac{\sum_{g=1}^{N}(x_i^{(g)} - \bar{x}_i)(x_j^{(g)} - \bar{x}_j)}{\sqrt{\sum_{g=1}^{N}(x_i^{(g)} - \bar{x}_i)^2}\sqrt{\sum_{g=1}^{N}(x_j^{(g)} - \bar{x}_j)^2}}, \tag{A1}$$

where N is the number of individuals in the training dataset, $C_{i,j}$ is the Pearson correlation coefficient between the i-th and j-th SNPs, $x_i^{(g)}$ is the genotype value of the g-th individual at the i-th SNP, and $\bar{x}_i$ is the mean genotype value at the i-th SNP across all individuals in the training dataset:

$$\bar{x}_i = \frac{1}{N}\sum_{g=1}^{N} x_i^{(g)}. \tag{A2}$$

This correlation matrix allows us to quantify the dependencies between SNPs and subsequently filter loci with an absolute correlation coefficient $|C_{i,j}| > 0.94$, ensuring that only a representative subset remains for further analysis. The procedure follows these steps:

- **Initialize:**
  - Define a set of remaining loci, R, containing all loci.
  - Define an initially empty set, S, to store selected independent loci.
- **Iterative selection:**
  - Randomly select a locus i from R and add it to S.
  - Identify all loci $j \in R$ that have an absolute correlation $|C_{i,j}| > 0.94$ with the selected locus i.
  - Remove these highly correlated loci from R.
  - Repeat the process (selecting a new locus from R, identifying correlated loci, and removing them) until no loci remain in R.
- **Output:** The final set S contains 1,164 loci.

This process filters out highly redundant loci (with pairwise correlations above 0.94), yielding a representative subset for further analysis.

Appendix B: Generation of simulated phenotype data

Simulated fitness values are generated using real genotype structures from 100,000 offspring of a budding yeast cross analyzed in Ref. [10]. The genotype data consists of 41,594 SNPs in total. To reduce redundancy, we select SNPs with a pairwise correlation of 94% or less, resulting in a filtered set of 1,164 SNPs (see Section A). From this subset, we pick either $L = 100$ or $L = 300$ for analysis. The simulated phenotype generation process is detailed below.

- **Temperature range.** Simulated fitness values are generated for the following temperature values:

$$T \in [23, 25, 27, 29, 31, 33, 35, 37]. \tag{B1}$$

- **Linear and epistatic effects.** The fitness values are modeled as a combination of linear effects and fourth-order epistatic interactions:

$$y^{(g)\text{syn}} = \beta_0 + \epsilon \sum_{l=1}^{L} \beta_l x_l^{(g)} + (1-\epsilon) \sum_{\substack{L \text{ nonzero} \\ \text{terms}}} \beta_{ijkl} x_i^{(g)} x_j^{(g)} x_k^{(g)} x_l^{(g)} + \eta, \tag{B2}$$

where β_0 is the intercept term, ϵ controls the proportion of linear vs. epistatic contributions, β_l represents the effect of the l-th locus, β_{ijkl} captures the fourth-order epistatic interaction effects, $x_l^{(g)}$ denotes the genotype of individual g at locus l, and η represents a noise term drawn from a Gaussian distribution. In Eq. (B2), fourth-order interaction terms are generated by selecting L unique sets of four loci at random.

- **Temperature dependence.** The genotype effect coefficients are modeled as quadratic functions of temperature:

$$\beta_l(T) = a_l(T-T_0)^2 + b_l(T-T_0) + c_l, \tag{B3}$$

$$\beta_{ijkl}(T) = a_{ijkl}(T-T_0)^2 + b_{ijkl}(T-T_0) + c_{ijkl}. \tag{B4}$$

Here, $T_0 = 30$ represents the reference temperature. The coefficients a, b, and c are sampled either from a normal distribution with mean $\mu = 0.5$ and standard deviation $\sigma = 0.5$:

$$a, b, c \sim \mathcal{N}(\mu = 0.5, \sigma = 0.5), \tag{B5}$$

or from an exponential distribution with a mean of 1:

$$a, b, c \sim \text{Exp}(\lambda = 1). \tag{B6}$$

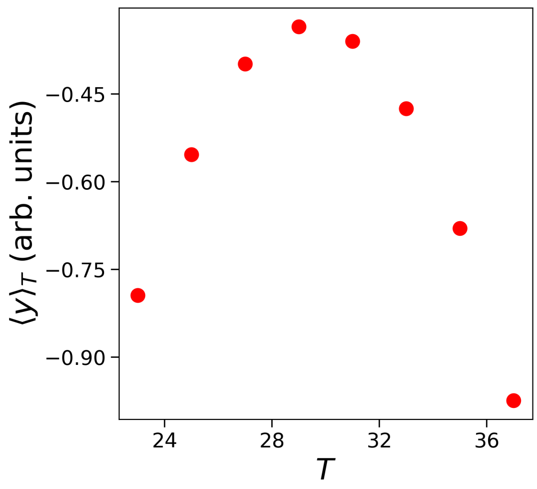

FIG. A1. Mean simulated fitness, $\langle y \rangle_T$, as a function of temperature T.

- **Scaling.** Fitness values are then scaled by a factor of 10^{-2} to ensure numerical stability and maintain meaningful ranges. The final scaled fitness value is given by:

$$y' = -10^{-2} y, \tag{B7}$$

where y is the original fitness value before scaling. The negative scaling is applied to ensure that the mean fitness exhibits a peak at $T = T_0$ rather than a minimum, aligning with biological expectations.

- **Noise addition.** To simulate measurement variability, random Gaussian noise is added to the generated fitness values. The noise η_α follows a normal distribution with mean 0 and a standard deviation set to 20% of the standard deviation of the fitness distribution in its respective environment:

$$\eta_\alpha \sim \mathcal{N}\left(0, 0.2 \cdot \sigma_\alpha\right), \tag{B8}$$

where σ_α is the standard deviation of fitness values in environment α.

Fig. A1 shows the mean simulated fitness values, $\langle y \rangle_T$, as a function of temperature T. The fitness exhibits a clear peak around $T = 30$, reflecting an optimal growth condition. This peaked behavior is biologically relevant, as many organisms exhibit temperature-dependent fitness landscapes, with growth rates declining at temperatures too far from their optimal range.

Appendix C: Implementation of the single-environment attention model

1. Model architecture

The implemented model consists of three sequential attention layers, each designed to learn genotype-phenotype mappings by attending to specific loci interactions [31]. To efficiently process high-dimensional genotype data, the model first applies a dimensionality reduction step before passing the embedded input through the initial attention layer. The key components of the architecture are as follows:

- **Input dimensionality and projection:**
  - The genotype data is initially represented in the range $[0, 1]$. We first transform it to the range $[-1, 1]$ using the transformation:

$$x_i^{(g)} \leftarrow 2x_i^{(g)} - 1, \tag{C1}$$

where $x_i^{(g)}$ is the genotype value for the g-th individual at the i-th SNP.

  - Each genotype of length L is converted into a one-hot embedding matrix $X^{(g)}$ of shape $L \times L$. The transformation follows:

$$X_{i,j}^{(g)} = \begin{cases} x_i^{(g)}, & \text{if } i = j \\ 0, & \text{otherwise} \end{cases}, \tag{C2}$$

where $X^{(g)}$ is a diagonal matrix with genotype values $x_i^{(g)}$ on the diagonal.
Implementation in PyTorch:
This transformation can be efficiently implemented in PyTorch as follows:

```
# Create a zero matrix of shape (batch_size,L,L):
one_hot_mini_batch_input = torch.zeros((batch_size, L,  L))

# Assign genotype values to the diagonal:
indices = torch.arange(L)
one_hot_mini_batch_input[:, indices, indices] = mini_batch_input.squeeze()
```

Here, `mini_batch_input.squeeze()` is a tensor of shape `batch_size` $\times L$, where each row corresponds to an individual, and each element in a row represents the genotype value at a given locus for that individual.

  - The model processes `batch_size` number of genotype matrices $X^{(g)}$, forming a three-dimensional tensor of shape `batch_size` $\times L \times L$.
  - Since the loci embedding vectors can be high-dimensional (e.g., $L = 1,164$ in the case of the BBQ budding yeast dataset), our architecture first applies a learned linear projection to map the input into a lower-dimensional latent space of size d. This transformation is performed as:

$$Z^{(g)} = X^{(g)} W_{\text{embed}}, \tag{C3}$$

where $W_{\text{embed}} \in \mathbb{R}^{L \times d}$ is a learnable projection matrix. For experimental data, we set $d = 12$, while for synthetic data, we use $d = 30$.

– Dimensionality reduction is not strictly necessary if computational resources and runtime are not a constraint. In such cases, we have also experimented with using the full-dimensional representation, where:

$$Z^{(g)} = X^{(g)}. \tag{C4}$$

However, unless specified otherwise, we will focus only on the case where dimensionality reduction is applied.

– Motivated by the series expansion results (see Section F), we append a constant value of 1 as an additional column to each transformed loci representation, effectively extending the embedding dimension from d to $d+1$. The transformation is given by:

$$\bar{Z}^{(g)} = \left[Z^{(g)} \;\; \mathbf{1} \right], \tag{C5}$$

where $Z^{(g)} \in \mathbb{R}^{L\times d}$ is the original embedding matrix for the g-th individual, and the appended column $\mathbf{1} \in \mathbb{R}^{L\times 1}$ consists of ones, resulting in an extended embedding matrix $\bar{Z}^{(g)} \in \mathbb{R}^{L\times(d+1)}$. This can be efficiently performed using `torch.cat` to concatenate a column of ones created with `torch.ones`.

- **Query-Key-Value mechanism and attention computation:**

– Each attention layer learns three parameter matrices—query, key, and value—for the following transformations:

$$Q^{(g)} = \bar{Z}^{(g)} W^Q, \quad Q^{(g)} \in \mathbb{R}^{L\times(d+1)}, \tag{C6}$$

$$K^{(g)} = \bar{Z}^{(g)} W^K, \quad K^{(g)} \in \mathbb{R}^{L\times(d+1)}, \tag{C7}$$

$$V^{(g)} = \bar{Z}^{(g)} W^V, \quad V^{(g)} \in \mathbb{R}^{L\times(d+1)}, \tag{C8}$$

where W^Q, W^K, and W^V are learnable projection matrices.

– The attention scores are computed using a scaled dot-product similarity between query and key matrices:

$$A^{(g)} = \mathrm{softmax}\left(Q^{(g)} {K^{(g)}}^T \right). \tag{C9}$$

– The final attended output is obtained by weighting the values using the computed attention scores:

$$Z'^{(g)} = A^{(g)} V^{(g)}. \tag{C10}$$

The attention scores are computed using `torch.matmul` for matrix multiplication, followed by `torch.softmax` for normalization.

– This process is repeated across three stacked attention layers, allowing the model to iteratively refine its understanding of genotype-phenotype relationships. The output of each layer serves as the input to the next layer. Specifically, the output of the first attention layer is denoted as $Z'^{(g)}$ and is used as input for the second layer, producing $Z''^{(g)}$, which in turn serves as input for the third layer, resulting in the final output $Z'''^{(g)}$.

- **Attention layers and final output computation:**

– The model consists of three sequential attention layers, each with its own set of learnable weight matrices.

– After the embeddings have been processed through the attention layers, the final representation $Z'''^{(g)}$ (obtained from the third attention layer) is used for prediction. The predicted phenotype $y^{(g)}_{\mathrm{pred}}$ for each individual g is given by:

$$y^{(g)}_{\mathrm{pred}} = \beta_0 + \sum_l \boldsymbol{\beta}_l \cdot \mathbf{z}'''^{(g)}_l. \tag{C11}$$

Here, $\boldsymbol{\beta}_l$ represents the weights associated with each embedding, and β_0 is the bias term.

- **Parameter initialization:**

- All learnable weight matrices are initialized using values drawn from a Gaussian distribution to ensure stable training. Specifically, each W is initialized as:

$$W \sim \mathcal{N}(0, \sigma^2), \tag{C12}$$

where σ varies depending on the specific architecture. In our work, σ ranges from 0.01 to 0.04, chosen based on empirical performance on the validation set to ensure stability and optimal convergence.

The key learnable weight matrices in the architecture include:

* **Embedding projection:** W_{embed}.
* **Query-Key-Value transformations:** W^Q, W^K, W^V — different for each layer.
* **Final linear transformation after attention:** β_0, $\boldsymbol{\beta}_l$.

2. Training and optimization

- **Data splitting:**
  - A total of 85% of the samples are allocated to training and validation, while the remaining 15% is reserved as the test dataset.
  - Within the training-validation set, 85% of the data is used for training, and 15% for validation.
- **Data normalization:**
  - Fitness values $y^{(g)}$ are standardized based on the mean and standard deviation of the training set to ensure numerical stability:

$$\texttt{train\_mean} = \frac{1}{N}\sum_{g=1}^{N} y_{\text{train}}^{(g)}, \tag{C13}$$

$$\texttt{train\_std} = \sqrt{\frac{1}{N}\sum_{g=1}^{N}\left(y_{\text{train}}^{(g)} - \texttt{train\_mean}\right)^2}. \tag{C14}$$

  - The training, validation, and test fitness values $y^{(g)}$ are normalized as:

$$\hat{y}^{(g)} = \frac{y^{(g)} - \texttt{train\_mean}}{\texttt{train\_std}}, \tag{C15}$$

where $\hat{y}^{(g)}$ denotes the normalized fitness values.

- **Training procedure:**
  - The model is trained using mini-batch gradient descent with a batch size of 64. During training, the first 64 samples are processed together in one batch, followed by the next 64, and so on, until the entire training dataset is covered in multiple iterations. The choice of 64 balances computational efficiency and gradient stability—small enough to fit within memory constraints while large enough to ensure smooth gradient updates and stable convergence.
  - Training is performed for more than 200 epochs, meaning the model iterated over the entire dataset at least 200 times. This ensures that the model has sufficient opportunities to learn complex genotype-phenotype relationships and reach a stable state where further training does not significantly improve performance.
  - The mean squared error (MSE) is used as the loss function, defined as:

$$\text{MSE} = \frac{1}{N}\sum_{i=1}^{N}(\hat{y}^{(g)} - \hat{y}_{\text{pred}}^{(g)})^2, \tag{C16}$$

where $\hat{y}^{(g)}$ represents the true normalized phenotype, $\hat{y}_{\text{pred}}^{(g)}$ is the predicted normalized phenotype, and N is the number of training samples in a mini-batch.

PyTorch syntax: `loss = torch.nn.MSELoss()`

- The Adam optimizer is used for training, with the learning rate depending on the genotype representation method. When dimensionality reduction is applied to loci embeddings, a learning rate of 0.001 is used, as it provides a good balance between convergence speed and stability, avoiding large oscillations in weight updates. However, when the original one-hot embedding of dimension L is used, a smaller learning rate of 0.0001 is chosen to ensure stable training and prevent large updates due to the high-dimensional input space.

  PyTorch syntax:

  * For dimensionality-reduced embeddings: `optimizer = torch.optim.Adam(attention_layer.parameters(), lr=0.001)`
  * For original one-hot embeddings of dimension L: `optimizer = torch.optim.Adam(attention_layer.parameters(), lr=0.0001)`

  The learning rate selection is based on empirical performance on the validation set, ensuring optimal convergence while preventing instability or slow training.

- Before each epoch, the dataset is shuffled to ensure that each mini-batch contains diverse samples, reducing bias and preventing the model from learning spurious correlations that might arise due to the order of data. Gradient updates are then applied iteratively on these mini-batches using backpropagation. The training process follows these key steps:

  * Zero the accumulated gradients from the previous iteration using: `optimizer.zero_grad()`
  * Compute the gradient of the loss function with respect to model parameters using backpropagation: `train_loss.backward()`
  * Update the model parameters using the optimizer: `optimizer.step()`

  These steps allow the model to iteratively minimize the loss function and improve generalization.

• **Validation and model selection:**

  - The validation dataset is used to monitor model performance at the end of each epoch, helping to assess how well the model generalizes to unseen data.
  - At the end of training, the epoch that achieved the highest R^2 on the validation set is identified, and the model parameters from that epoch are saved as the best-performing version.
  - This saved version is then restored and used for the final evaluation on the test dataset.

• **Test performance evaluation:**

  - After training, the model is evaluated on the test dataset to assess its generalization ability.

• **Memory optimization:**

  - To prevent memory overload, phenotype predictions are processed in smaller batches, specifically in chunks of 100 segregants. This approach ensures efficient memory usage while maintaining computational speed.
  - GPU memory usage is actively managed by clearing unused tensors with `torch.cuda.empty_cache()` between computations. This helps free up allocated memory, reducing the risk of out-of-memory errors and ensuring smoother model execution.

Appendix D: Multi-environment attention-based model

This architecture differs from the single-environment attention model in two key aspects: the embedding representation and the mini-batch selection strategy. Here, we focus on these differences.

1. Encoding genotypes and environments

In the multi-environment model, each environmental condition is represented by a one-hot encoded vector of dimension equal to the total number of environments, E. The entry corresponding to the environment's index is set to 1, while all other entries are 0. These environmental embeddings are processed alongside the genotype embeddings within the attention-based architecture, allowing the model to learn environment-specific phenotypic effects without explicit concatenation.

To ensure compatibility within the attention mechanism:

- The d-dimensional loci embeddings $Z^{(g)}$ (see Eq. (C3)) are padded with E zeros on the right:

$$\bar{Z}^{(g)} = \left[Z^{(g)} \ \ \mathbf{0}_{L\times E}\right], \quad \bar{Z}^{(g)} \in \mathbb{R}^{L\times(d+E)}. \tag{D1}$$

If no dimensionality reduction is applied (i.e., the full loci embedding size L is used), the loci embeddings are padded as:

$$\bar{Z}^{(g)} = \left[Z^{(g)} \ \ \mathbf{0}_{L\times E}\right], \quad \bar{Z}^{(g)} \in \mathbb{R}^{L\times(L+E)}. \tag{D2}$$

- The E-dimensional one-hot encoded environment embeddings $\mathbf{e}_\alpha$ are padded with d zeros on the left:

$$\bar{\mathbf{e}}_\alpha = \left[\mathbf{0}_{1\times d} \ \ \mathbf{e}_\alpha\right], \quad \bar{\mathbf{e}}_\alpha \in \mathbb{R}^{1\times(d+E)}. \tag{D3}$$

If no dimensionality reduction is applied (i.e., the full loci embedding size L is used), the environment embeddings are padded as:

$$\bar{\mathbf{e}}_\alpha = \left[\mathbf{0}_{1\times L} \ \ \mathbf{e}_\alpha\right], \quad \bar{\mathbf{e}}_\alpha \in \mathbb{R}^{1\times(L+E)}. \tag{D4}$$

- A constant feature of 1 is appended to both the loci and environment embeddings at the rightmost position:

$$\tilde{Z}^{(g)} = \left[\bar{Z}^{(g)} \ \ \mathbf{1}_{L\times 1}\right], \quad \tilde{Z}^{(g)} \in \mathbb{R}^{L\times(d+E+1)}. \tag{D5}$$

$$\tilde{\mathbf{e}}_\alpha = \left[\bar{\mathbf{e}}_\alpha \ \ 1\right], \quad \tilde{\mathbf{e}}_\alpha \in \mathbb{R}^{1\times(d+E+1)}. \tag{D6}$$

If no dimensionality reduction is applied, the final representations become:

$$\tilde{Z}^{(g)} = \left[\bar{Z}^{(g)} \ \ \mathbf{1}_{L\times 1}\right], \quad \tilde{Z}^{(g)} \in \mathbb{R}^{L\times(L+E+1)}. \tag{D7}$$

$$\tilde{\mathbf{e}}_\alpha = \left[\bar{\mathbf{e}}_\alpha \ \ 1\right], \quad \tilde{\mathbf{e}}_\alpha \in \mathbb{R}^{1\times(L+E+1)}. \tag{D8}$$

This alignment ensures that both genotype and environment representations share the same dimensional scale, $d+E+1$ (or $L+E+1$), during attention computations. This structured encoding allows the model to dynamically infer gene-environment interactions.

2. Training strategy

To effectively capture genotype-phenotype relationships across multiple environments, the model is trained using mini-batch stochastic gradient descent. We ensure that each mini-batch includes samples from all environments, promoting cross-environment learning and enhancing the model's ability to generalize across diverse conditions. The training setup follows these principles:

Balanced representation of environments: Each mini-batch includes an equal number of individuals from all environments, preventing any single environment from dominating the training process. This ensures that the model learns environment-specific effects in a balanced manner. Given that each mini-batch contains $N_{\text{ind},\alpha} = 4$ individuals per environment and there are $E = 18$ environments, the total `batch_size` is given as:

$$\texttt{batch\_size} = \sum_{\alpha=1}^{E} N_{\text{ind},\alpha} = \sum_{\alpha=1}^{18} 4 = 72. \tag{D9}$$

This setup prevents the model from developing biases toward specific environments and ensures that predictions remain stable rather than fluctuating unpredictably as the mini-batch changes across training iterations.

Cross-talking between environments: Including individuals from all environments in each mini-batch allows the model to simultaneously process information from different environmental conditions and update its parameters based on a comprehensive view of genotype-phenotype interactions across environments.

Since each mini-batch contains E environments, with $N_{\text{ind},\alpha}$ individuals per environment, the batch-wise loss function aggregates gradients across environments becomes:

$$\mathcal{L}_{\text{batch}} = \frac{1}{E}\sum_{\alpha=1}^{E}\mathcal{L}_{\alpha}, \tag{D10}$$

where $\mathcal{L}_{\alpha}$ is the loss computed for $N_{\text{ind},\alpha}$ individuals from environment α. During backpropagation, the gradient updates are computed jointly:

$$\theta \leftarrow \theta - \eta\frac{1}{E}\sum_{\alpha=1}^{E}\nabla_{\theta}\mathcal{L}_{\alpha}, \tag{D11}$$

where: θ represents the model parameters, η is the learning rate, and $\nabla_{\theta}\mathcal{L}_{\alpha}$ is the gradient contribution from environment α. This ensures that individuals from different environments influence shared model parameters, allowing information from one environment to shape predictions in another, even without direct cross-attention.

The remainder of the training procedure, as well as the validation and testing processes, are identical to those described for the single-environment attention model in Section C.

Appendix E: Transfer learning

In scenarios where data for a specific environment is sparse, the multi-environment attention-based model can leverage correlations across environments to improve predictions in the underrepresented environment. This approach enables the model to infer missing phenotypic values by exploiting shared genetic and environmental structures.

Let there be a total of E environments, each represented as a one-hot vector $\mathbf{e}_{\alpha} \in \mathbb{R}^{E}$. In our work, there are eight temperatures (see Eq. (B1)), so $E = 8$. For the synthetic data, we use $d = 30$, and thus $d > E$. To match the dimensionality of the loci embeddings, the environment tokens are padded with zeros such that the rightmost $d - E$ values are set to zero. This ensures compatibility within the attention mechanism while maintaining a distinct representation for each environment. The padded environment embedding is defined as:

$$\bar{\mathbf{e}}_{\alpha} = \begin{bmatrix}\mathbf{e}_{\alpha} & \mathbf{0}_{1\times(d-E)}\end{bmatrix}, \quad \bar{\mathbf{e}}_{\alpha} \in \mathbb{R}^{1\times d}. \tag{E1}$$

A constant feature of 1 is then appended to maintain compatibility with loci embeddings (Eq. (C5)):

$$\tilde{\mathbf{e}}_{\alpha} = \begin{bmatrix}\bar{\mathbf{e}}_{\alpha} & 1\end{bmatrix}, \quad \tilde{\mathbf{e}}_{\alpha} \in \mathbb{R}^{1\times(d+1)}. \tag{E2}$$

This transformation ensures that both genotype and environment embeddings have the same dimension $d+1$, allowing them to be processed seamlessly within the attention mechanism.

To simulate data sparsity in a single environment, we randomly select one environment—let us call it the environment α^*—and introduce missing values. This is done by randomly selecting $N_{\alpha^*} - M_{\alpha^*}$ fitness values within this environment and setting them to `NaN` (Not a Number). In computing, `NaN` is a special placeholder used to represent missing, undefined, or unrepresentable numerical values. Here, N_{α^*} represents the total number of training samples in environment α^*, and M_{α^*} denotes the number of samples available to the model during training. The training samples from all other environments remain fully available.

The mini-batch structure is designed to maintain a balanced representation across environments. Each batch consists of four individuals per environment (see Eq. (D9)). However, when computing the loss function (see Eq. (D10)), individuals with NaN fitness values are discarded, ensuring that only available data contribute to parameter updates.

This setup allows the underrepresented environment, α^*, to interact with all other environments during every mini-batch processing, facilitating cross-environment learning.

The remainder of the training procedure, as well as the validation and testing processes, are identical to those described for the single-environment attention model in Section C.

Appendix F: Series expansions of an attention layer

In this section, we derive a series expansion for the output of a single attention layer, considering the case where one-hot encoded loci vectors are fed directly into the layer without any dimensionality reduction. We use the following notations throughout:

- $\mathbf{z}_i$: Embedding vector for the ith locus.
- $\mathbf{q}_i$: Query vector for the ith locus.

- $\mathbf{k}_i$: Key vector for the ith locus.
- $\mathbf{v}_i$: Value vector for the ith locus.
- W^Q, W^K, W^V: Query, key, and value projection matrices, respectively.
- C^i: Coefficients of the i-th order interaction.
- β: Regression coefficients for the attended vectors.
- $x_i \in \{+1, -1\}$: SNP value associated with the ith locus.

We start by considering the scenario where the one-hot encoding of the loci is directly provided to the attention layer without any prior dimensionality reduction.

The νth component of the value vector $\mathbf{v}_j$, denoted by $v_{j\nu}$, is computed as follows:

$$v_{j\nu} = \sum_m z_{jm}\, W^V_{m\nu}. \tag{F1}$$

Since the one-hot embedding implies that $z_{jm} = \delta_{jm}\, x_j$ (where δ_{jm} is the Kronecker delta), this expression simplifies to:

$$v_{j\nu} = \sum_m \delta_{jm}\, x_j\, W^V_{m\nu} = x_j\, W^V_{j\nu}. \tag{F2}$$

Similarly, we obtain $q_{im} = x_i\, W^Q_{im}$ and $k_{jm} = x_j\, W^K_{jm}$.

The dot product between the query vector $\mathbf{q}_i$ and the key vector $\mathbf{k}_j$ is given by:

$$\mathbf{q}_i \cdot \mathbf{k}_j = \sum_m q_{im}\, k_{jm}. \tag{F3}$$

Given that $q_{im} = x_i\, W^Q_{im}$ and $k_{jm} = x_j\, W^K_{jm}$, we obtain:

$$\mathbf{q}_i \cdot \mathbf{k}_j = \sum_m x_i\, W^Q_{im}\, x_j\, W^K_{jm} = x_i\, x_j\, \alpha_{ij}, \tag{F4}$$

where we define

$$\alpha_{ij} = \sum_m W^Q_{im}\, W^K_{jm}. \tag{F5}$$

The predicted fitness, denoted by y_{pred}, is given by:

$$y_{\text{pred}} = \sum_{i,\nu} \left[\sum_j \frac{e^{\mathbf{q}_i \cdot \mathbf{k}_j}}{\sum_m e^{\mathbf{q}_i \cdot \mathbf{k}_m}}\, v_{j\nu} \right] \beta_{i\nu} + \beta_0. \tag{F6}$$

We expand the softmax term as a Taylor series:

$$\frac{e^{\mathbf{q}_i \cdot \mathbf{k}_j}}{\sum_m e^{\mathbf{q}_i \cdot \mathbf{k}_m}} = \frac{1 + \mathbf{q}_i \cdot \mathbf{k}_j + \frac{(\mathbf{q}_i \cdot \mathbf{k}_j)^2}{2!} + \frac{(\mathbf{q}_i \cdot \mathbf{k}_j)^3}{3!} + \cdots}{\sum_m \left(1 + \mathbf{q}_i \cdot \mathbf{k}_m + \frac{(\mathbf{q}_i \cdot \mathbf{k}_m)^2}{2!} + \frac{(\mathbf{q}_i \cdot \mathbf{k}_m)^3}{3!} + \cdots \right)}. \tag{F7}$$

Inserting $\mathbf{q}_i \cdot \mathbf{k}_j = x_i\, x_j\, \alpha_{ij}$ from Eq. (F4), we have:

$$\frac{e^{\mathbf{q}_i \cdot \mathbf{k}_j}}{\sum_m e^{\mathbf{q}_i \cdot \mathbf{k}_m}} = \frac{1 + x_i\, x_j\, \alpha_{ij} + \frac{\alpha_{ij}^2}{2} + x_i\, x_j\, \frac{\alpha_{ij}^3}{6} + \cdots}{\sum_m \left(1 + x_i\, x_m\, \alpha_{im} + \frac{\alpha_{im}^2}{2} + x_i\, x_m\, \frac{\alpha_{im}^3}{6} + \cdots \right)}. \tag{F8}$$

We can express the series in terms of hyperbolic functions by recognizing that:

$$1 + x_i\, x_j\, \alpha_{ij} + \frac{\alpha_{ij}^2}{2} + x_i\, x_j\, \frac{\alpha_{ij}^3}{6} + \cdots = \frac{e^{\alpha_{ij}} + e^{-\alpha_{ij}}}{2} + x_i\, x_j\, \frac{e^{\alpha_{ij}} - e^{-\alpha_{ij}}}{2}. \tag{F9}$$

Thus, the expression becomes:

$$\frac{e^{\mathbf{q}_i\cdot\mathbf{k}_j}}{\sum_m e^{\mathbf{q}_i\cdot\mathbf{k}_m}} = \frac{\beta_{ij}^+ + x_i\, x_j\, \beta_{ij}^-}{\sum_m \left(\beta_{im}^+ + x_i\, x_m\, \beta_{im}^-\right)}, \tag{F10}$$

where we have defined

$$\beta_{ij}^{\pm} = \frac{e^{\alpha_{ij}} \pm e^{-\alpha_{ij}}}{2}. \tag{F11}$$

Let

$$g_i^+ = \sum_m \beta_{im}^+ \quad \text{and} \quad h_{ij}^{\pm} = \frac{\beta_{ij}^{\pm}}{g_i^+}. \tag{F12}$$

With these definitions, the softmax expression simplifies to:

$$\frac{e^{\mathbf{q}_i\cdot\mathbf{k}_j}}{\sum_m e^{\mathbf{q}_i\cdot\mathbf{k}_m}} = \frac{h_{ij}^+ + x_i\, x_j\, h_{ij}^-}{1 + x_i \sum_m x_m\, h_{im}^-}. \tag{F13}$$

Now, substituting the above and the expression for $v_{j\nu}$ from Eq. (F2) into Eq. (F6), we obtain:

$$y_{\text{pred}} = \sum_{i,j,\nu} \frac{x_j\, h_{ij}^+ + x_i\, h_{ij}^-}{1 + x_i \sum_m x_m\, h_{im}^-}\, W_{j\nu}^V\, \beta_{i\nu} + \beta_0. \tag{F14}$$

Expanding the denominator as a series, we have:

$$\frac{1}{1 + x_i \sum_m x_m\, h_{im}^-} \approx 1 - x_i \sum_m x_m\, h_{im}^- + \sum_{m,m'} x_m\, x_{m'}\, h_{im}^-\, h_{im'}^- - x_i \sum_{m,m',m''} x_m\, x_{m'}\, x_{m''}\, h_{im}^-\, h_{im'}^-\, h_{im''}^- + \cdots. \tag{F15}$$

Thus, the predicted fitness can be expressed as:

$$y_{\text{pred}} = \sum_{i,j,\nu} W_{j\nu}^V\, \beta_{i\nu}\, \left(x_j\, h_{ij}^+ + x_i\, h_{ij}^-\right) \left(1 - x_i \sum_m x_m\, h_{im}^- + \sum_{m,m'} x_m\, x_{m'}\, h_{im}^-\, h_{im'}^- - \cdots\right) + \beta_0. \tag{F16}$$

This expansion can be rearranged to show:

$$y_{\text{pred}} = C^0 + \sum_i C_i^1\, x_i + \sum_{i,j,k} C_{ijk}^3\, x_i\, x_j\, x_k + \cdots. \tag{F17}$$

Notice that this series does not include explicit pairwise interactions between loci (i.e., terms like $x_i\, x_j$ do not appear on their own).

Changing embedding to incorporate pairwise interactions

We consider a simple modification to the embedding—appending an additional element 1 at the end of the one-hot vectors. With this change,

$$z_{i\nu} = \delta_{i\nu}\, x_i + \delta_{L+1,\nu},$$

so that the new embedding is $(L+1)$-dimensional. The value vector is then given as:

$$\begin{aligned} v_{j\nu} &= \sum_m z_{jm}\, W^V_{m\nu} \\ &= \sum_m \left(\delta_{jm}\, x_j + \delta_{L+1,m}\right) W^V_{m\nu} \\ &= x_j\, W^V_{j\nu} + W^V_{L+1,\nu}, \end{aligned} \tag{F18}$$

and the dot product $\mathbf{q}_i \cdot \mathbf{k}_j$ can be obtained as

$$\begin{aligned} \mathbf{q}_i \cdot \mathbf{k}_j &= \sum_m q_{im}\, k_{jm} \\ &= \sum_m \left(x_i\, W^Q_{im} + W^Q_{L+1,m}\right) \left(x_j\, W^K_{jm} + W^K_{L+1,m}\right) \\ &= x_i\, x_j\, \alpha_{ij} + x_i\, \beta_i + x_j\, \gamma_j + s, \end{aligned} \tag{F19}$$

where

$$\begin{aligned} \alpha_{ij} &= \sum_m W^Q_{im}\, W^K_{jm}, \quad & \eta_i &= \sum_m W^Q_{im}\, W^K_{L+1,m}, \\ \gamma_j &= \sum_m W^Q_{L+1,m}\, W^K_{jm}, \quad & s &= \sum_m W^Q_{L+1,m}\, W^K_{L+1,m}. \end{aligned} \tag{F20}$$

Without the softmax, the fitness y_{pred} is given by:

$$y_{\text{pred}} = \sum_{i,\nu} \left[\sum_j \left(\mathbf{q}_i \cdot \mathbf{k}_j\right)\, v_{j\nu} \right] \beta_{i\nu} + \beta_0. \tag{F21}$$

Substituting Eqs. (F18) and (F19) into Eq. (F21), we obtain:

$$\begin{aligned} y_{\text{pred}} =& \sum_{i,j,\nu} \left(x_i\, x_j\, \alpha_{ij} + x_i\, \eta_i + x_j\, \gamma_j + s\right) \left(x_j\, W^V_{j\nu} + W^V_{L+1,\nu}\right) \beta_{i\nu} + \beta_0 \\ =& \, C^0 + \sum_i C^1_i\, x_i + \sum_{i,j} C^2_{ij}\, x_i\, x_j, \end{aligned} \tag{F22}$$

where

$$\begin{aligned} C^0 &= \beta_0 + \sum_{i,j,\nu} \beta_{i\nu} \left(\gamma_j\, W^V_{j\nu} + s\, W^V_{L+1,\nu}\right), \\ C^1_i &= \sum_{\nu,j} \left\{ \beta_{i\nu} \left(\alpha_{ij}\, W^V_{j\nu} + \eta_i\, W^V_{L+1,\nu}\right) + \beta_{j\nu} \left(\gamma_i\, W^V_{L+1,\nu} + s\, W^V_{i\nu}\right) \right\}, \\ C^2_{ij} &= \sum_\nu \beta_{i\nu} \left(\alpha_{ij}\, W^V_{L+1,\nu} + \eta_i\, W^V_{j\nu}\right). \end{aligned}$$

Even without the softmax, we observe an explicit pairwise interaction term. With the softmax applied, higher-order terms naturally emerge. This motivates us to always append a constant value of 1 to the end of all embedding vectors.

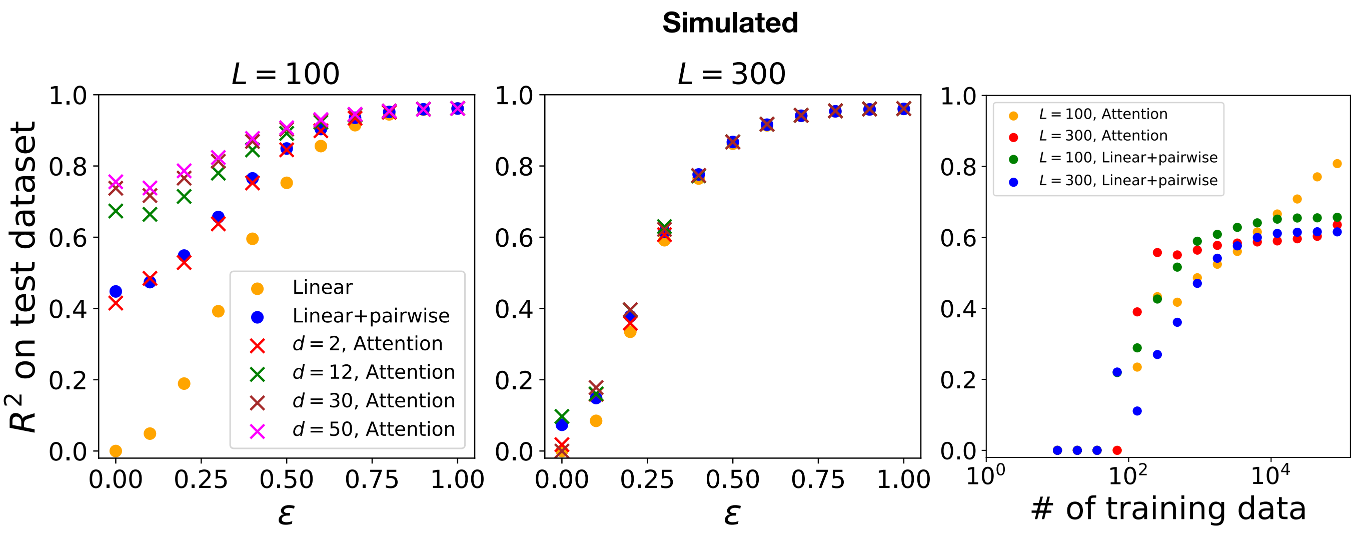

FIG. A2. **Performance comparison of models for exponentially distributed coefficients β with varying embedding dimensions d and number of loci L.** The left and center panels show R^2 values on the test dataset for $L = 100$ and $L = 300$, respectively, across different epistasis strengths ϵ. The right panel illustrates R^2 performance at $\epsilon = 0.3$ as a function of training dataset size, using an embedding dimension of $d = 30$ for the attention-based models.

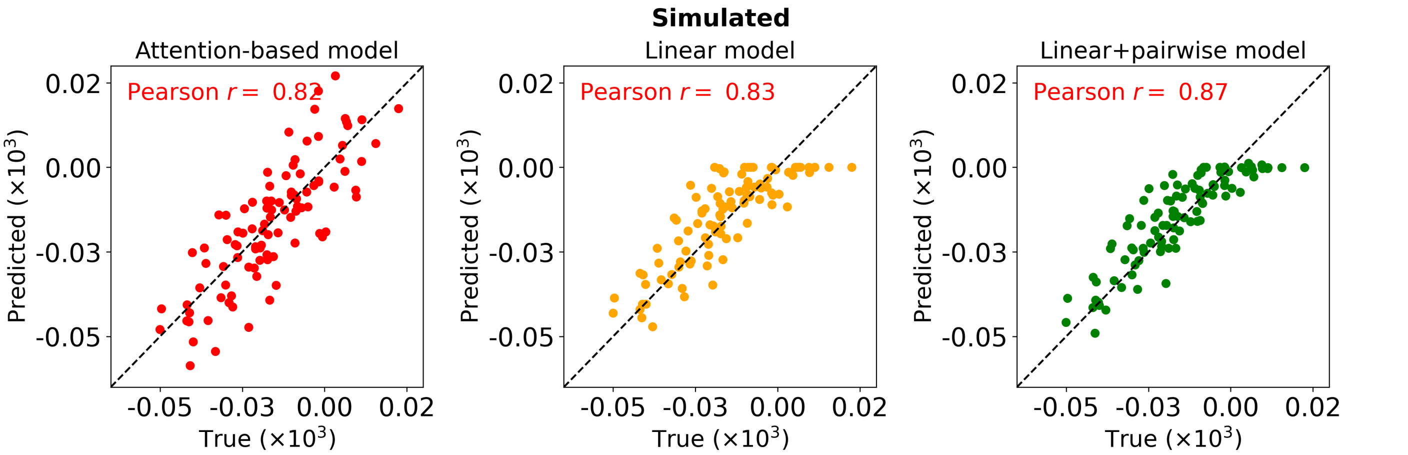

FIG. A3. **Comparison of predicted effect sizes for Gaussian-distributed coefficients with $L = 100$ and $\epsilon = 0.3$.** We compare the predicted effect sizes, averaged over genotype backgrounds, on the test dataset across three models: attention-based model with $d = 30$ (left), linear model (middle), and linear+pairwise model (right). Each plot shows the correlation between the predicted and true effect sizes, with the respective Pearson correlation coefficients indicated.

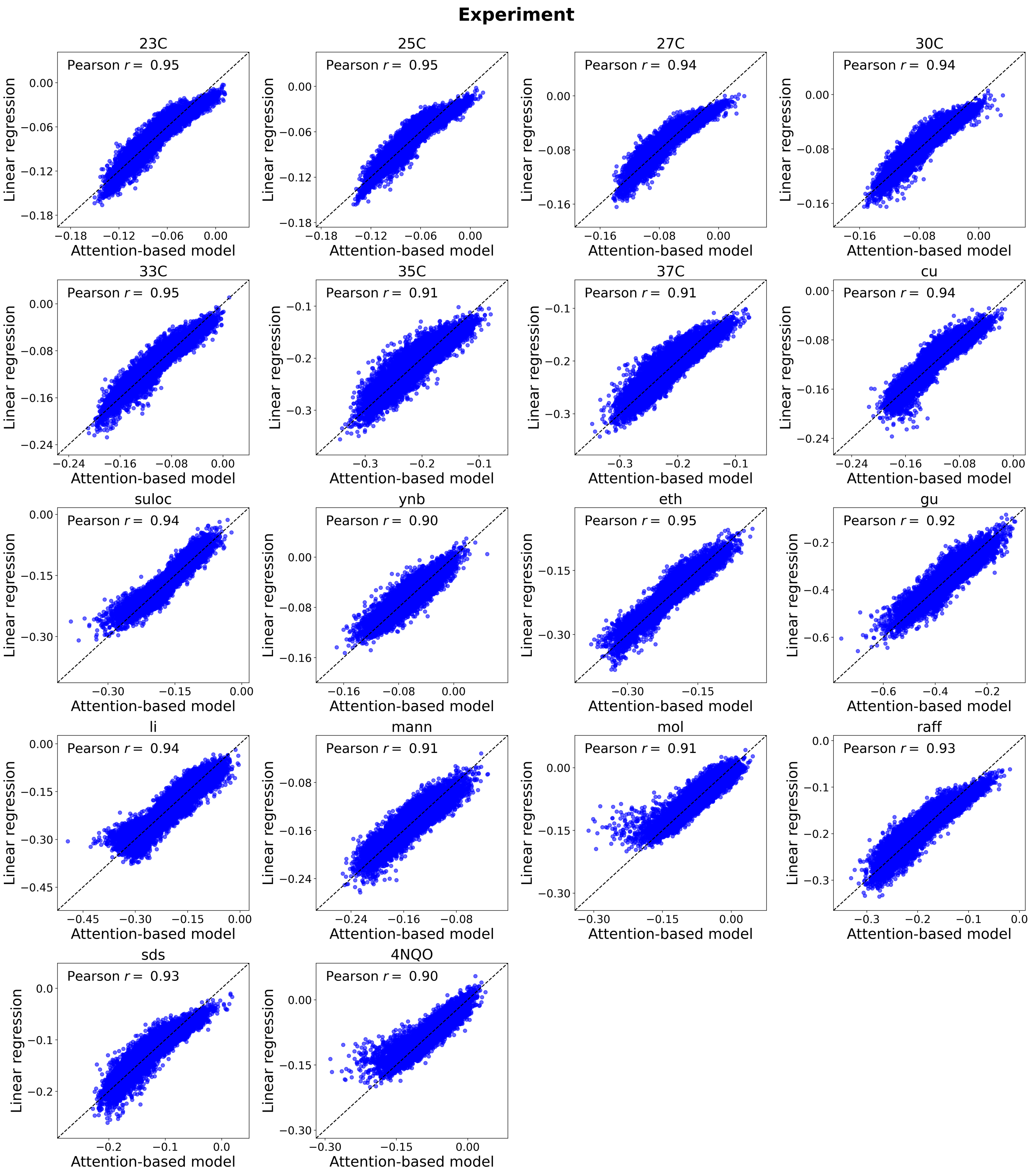

FIG. A4. **Comparison of fitness predictions from linear regression and a single-environment attention-based model across multiple environments.** Each subplot corresponds to one of eighteen different media conditions (indicated in the subplot titles) and shows the predicted fitness values for all test-set individuals. The y-axis represents the linear regression predictions, while the x-axis shows those from the attention-based model. Pearson correlation coefficients are shown in each subplot.

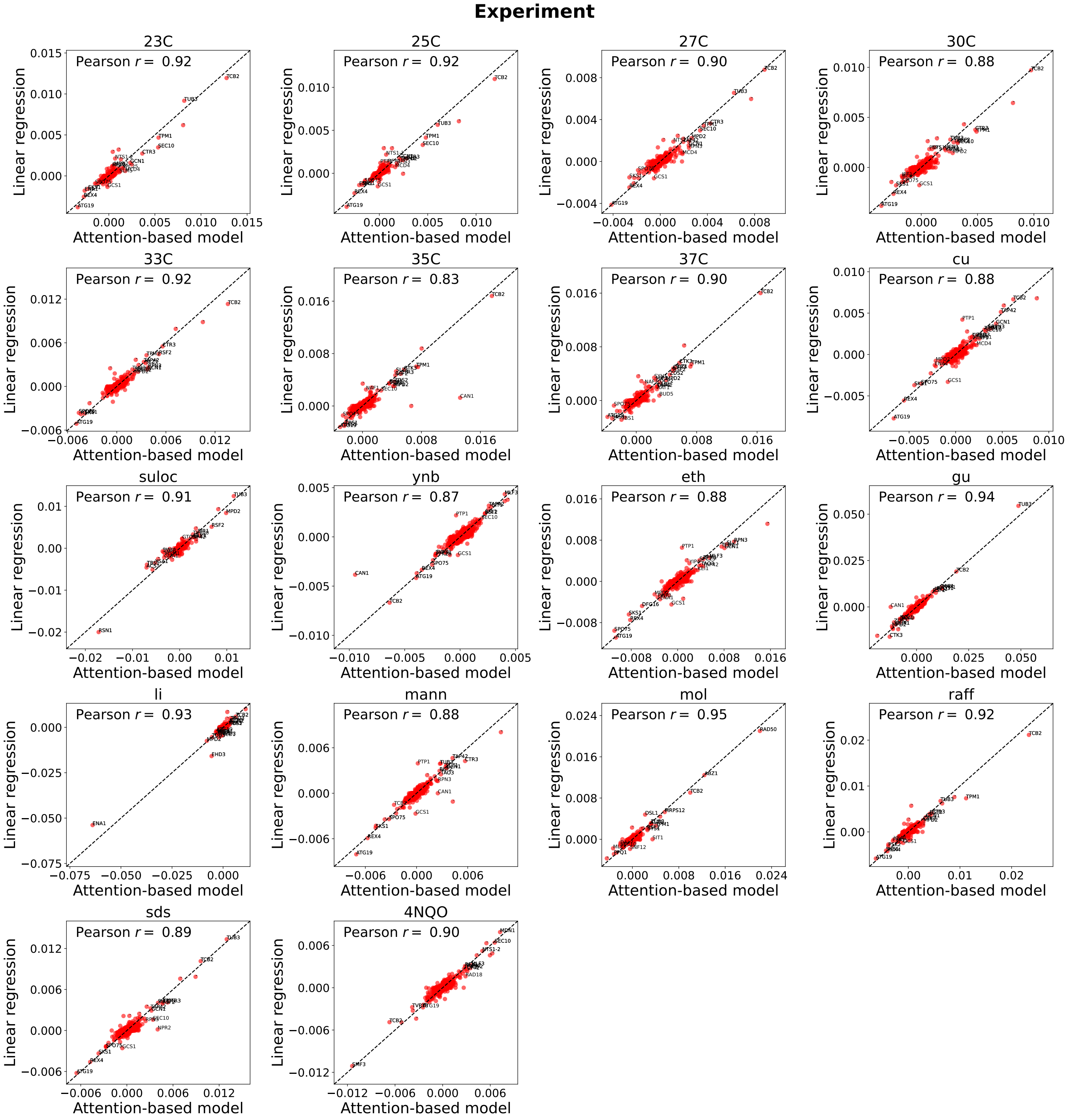

FIG. A5. **Comparison of effect sizes predicted by linear regression and a single-environment attention-based model under different conditions.** Each subplot corresponds to a different environmental condition and compares effect sizes predicted by the two models. The loci with the 20 largest effect sizes are annotated. Pearson correlation coefficients are shown in each subplot.

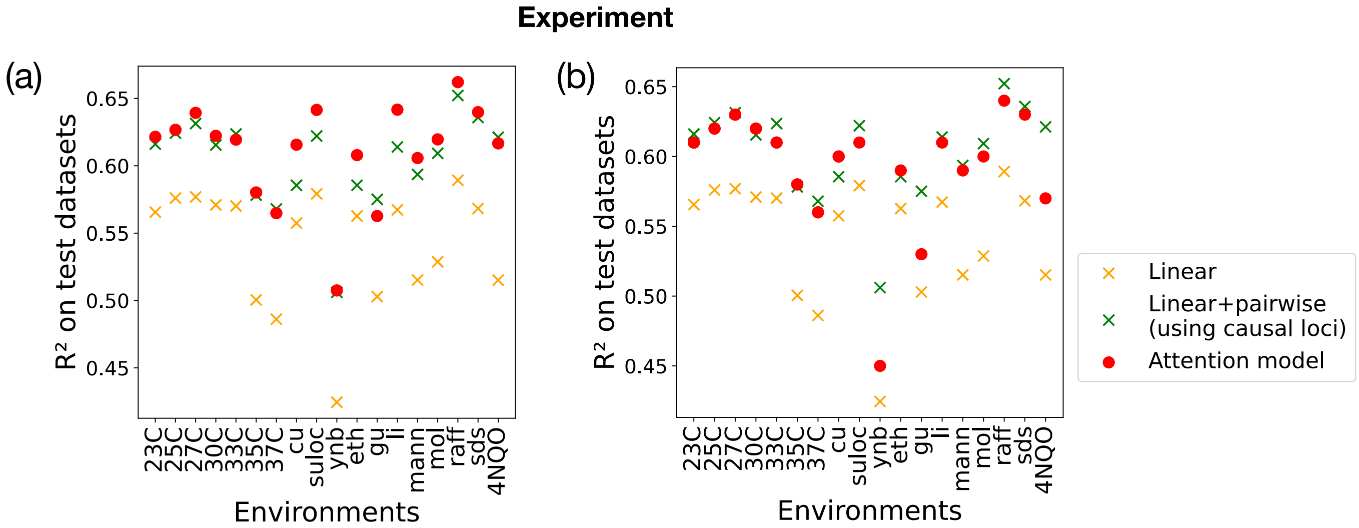

FIG. A6. **Performance comparison of attention-based architectures with one-hot embeddings for loci and environment.** The R^2 values on test datasets are shown for the linear model, the linear+pairwise model (using causal loci), and the attention-based model across various environments. (a) Single-environment attention architecture. (b) Multi-environment attention architecture. Both architectures use two attention layers.

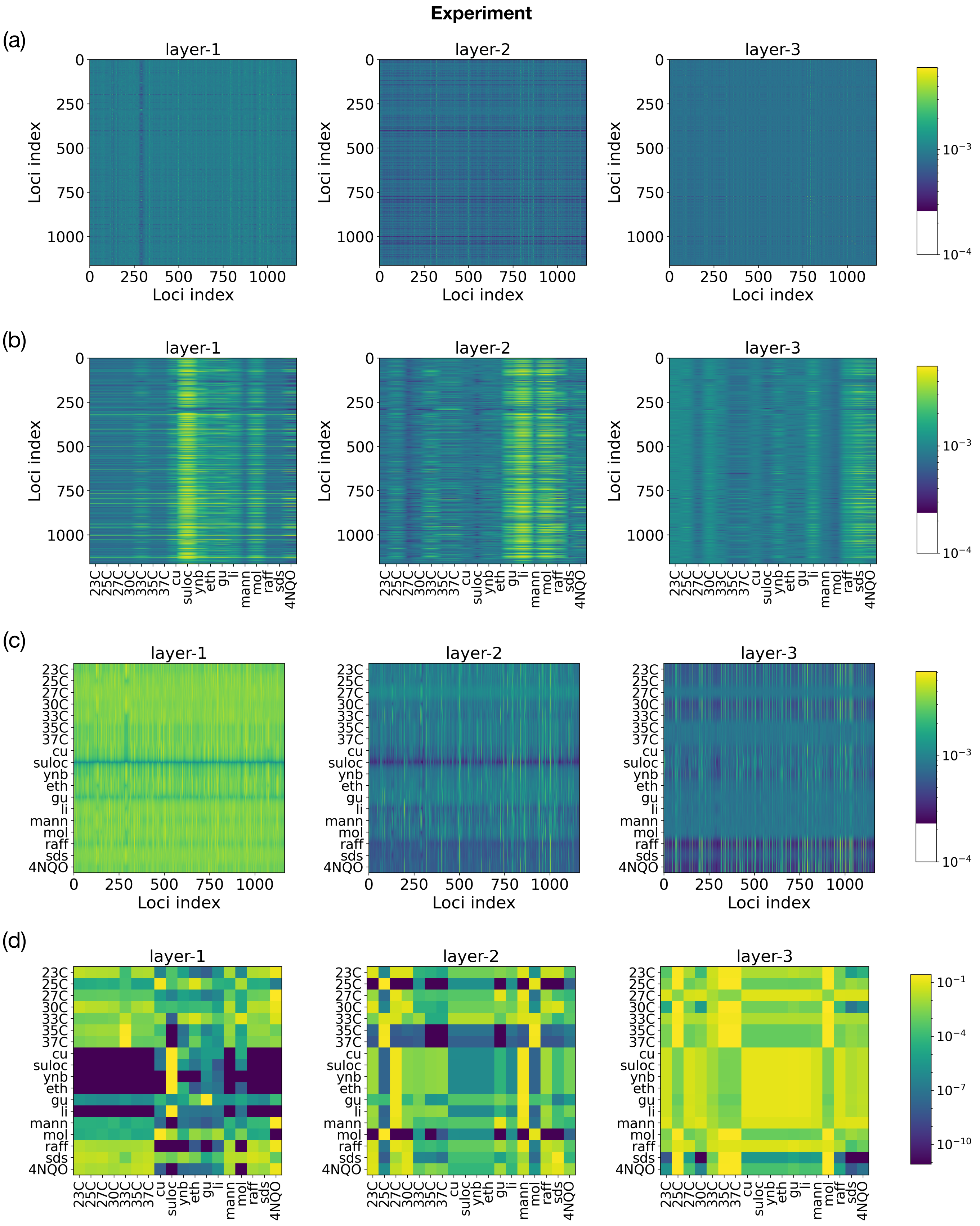

FIG. A7. **Heatmaps of attention scores across three successive layers in the multi-environment architecture with $d = 12$.** The scores are computed using Eq. (C9) and averaged across all test segregants. The horizontal axis represents key vectors, and the vertical axis represents query vectors. Panels (a)–(d) show locus–locus, environment–locus, environment–locus, and environment–environment attention maps, respectively. Note that the key and query vectors for environments are independent of the individuals.
